# Emotional regulation and music engagement: a meta-analytic synthesis

**DOI:** 10.3389/fpsyg.2026.1851432

**Published:** 2026-09-03

**Authors:** Yuquan Feng, Hao Wang, Yelin Tan, Fuhong Wang

**Affiliations:** 1 Music Education and Psychology, College of Music, Sichuan Normal University, Chengdu, Sichuan, China; 2 Vocal Performance and Music Education, College of Music, Sichuan Normal University, Chengdu, Sichuan, China; 3 Cross-cultural Studies and Music, College of Humanities, Central South University, Changsha, Hunan, China; 4 Music History and Theory, School of Humanities and Arts, Southwestern University of Finance and Economics, Chengdu, Sichuan, China

**Keywords:** adolescents, children, clinical populations, emotion regulation, mood, music therapy, psychological well-being, stress reduction

## Abstract

**Background:**

The use of music-based interventions has grown to support emotional regulation, mood improvement, stress relief, and psychological well-being in general, pediatric, and clinical populations. The research shows increasing evidence but maintains gaps in understanding how the treatments work across different populations.

**Objective:**

The study aims to synthesize existing research on music-based interventions that help people regulate their emotions, maintain their mood, and manage stress across various groups through a meta-analysis to determine the overall impact of these interventions.

**Methods:**

The researchers conducted a comprehensive literature search to identify articles published between 2007 and 2026 in the PubMed, Scopus, and Web of Science databases. The study included research on music-based treatments for emotional control and mood improvement, stress management, and psychological wellness across general, pediatric, adolescent, and clinical populations. The researchers extracted raw depression or mood scores from the data and analyzed them using a random-effects model with inverse-variance weighting. The researchers assessed heterogeneity using the I^2^ statistic and Cochran’s Q test.

**Results:**

A total of 46 studies were included in the research. The general population showed depression results of 18.25 (95% CI: 17.76–18.75) through music interventions, which included emotion regulation mobile apps, mindfulness-based music therapy, and personal music listening, according to 13 studies that involved 1,041 participants. The eight studies, with 497 participants, found that group music therapy, impromptu music therapy, and music-induced embodied emotion regulation produced a score of 18.12 (95% CI: 17.17–19.07), with high heterogeneity (I^2^ = 75%, *p* < 0.01). The clinical and special populations research showed that ADHD, TBI, cancer, substance use, and transplant patient interventions produced a MRAW of 19.58 (95% CI: 19.03–20.14) with moderate heterogeneity (I^2^ = 52%, *p* = 0.02). The seven studies with 497 participants that examined stress reduction, coping, and psychological well-being found an MRAW score of 18.80 (95% CI: 17.82–19.78), indicating significant heterogeneity (I^2^ = 80%, *p* < 0.01). The results show that music-based interventions lead to moderate emotional improvements while clinical populations exhibit slightly elevated initial depressive levels.

**Conclusion:**

Music-based interventions are effective tools that help people from diverse backgrounds regulate their emotions, improve mood, reduce stress, and achieve better psychological outcomes. The interventions yield consistent positive results, with varying levels of effectiveness across clinical and special populations. Future research should investigate the optimal types of interventions, their duration, and delivery methods to maximize emotional and psychological outcomes.

## 1 Introduction

People have used music throughout history because it functions as an emotional expression tool and a psychological control mechanism. People from different cultures and throughout history have used music to create artistic works, enjoy leisure activities, change their emotional states, handle stress, and build social connections (Shan and Qi, 2024; Tang et al., 2025; Wang et al., 2024). Psychologists recognize music as a sophisticated stimulus that activates multiple brain systems, cognitive functions, and emotional responses. Music has been shown to change emotional states, improve coping abilities, and help people control their emotions, making it useful for both medical and non-medical applications (Lyu et al., 2026; Qin et al., 2025; Tang et al., 2026).

Emotion regulation enables people to control their emotional experiences by choosing which feelings to experience at which moments and which emotions to show. People who use ineffective methods to control their emotions will develop multiple mental health problems, which include depression, anxiety, and stress-related disorders (Chin and Rickard, 2012; Fan and Zhao, 2025; Lin et al., 2026). People who can handle their emotions successfully will develop resilience, improve their mental health, and adapt to life’s challenges. Researchers are conducting studies on music-based treatments because they believe these treatments can help people manage their emotions through mechanisms such as maintaining focus, regulating energy levels, and altering thought patterns. Music helps people better understand their emotions while providing enjoyable experiences that help them learn to identify their feelings and express and manage them in healthy ways (Chong et al., 2024; Huang and Xu, 2025; Qiao et al., 2026a).

Music enables people to manage their emotions while also helping them achieve better mood and overall mental health outcomes. Music can create positive emotions in listeners while helping them relax and decreasing their negative feelings. Personalized music listening has been demonstrated to decrease stress levels and improve mood and subjective well-being in both healthy adults and vulnerable populations (Chu et al., 2025; Saarikallio, 2011). Participatory music activities, which include singing, drumming, improvisation, and group music therapy, provide people with social connections and mastery experiences that help them build emotional resilience. People who deal with increased psychological stress should use these experiences, which are suitable for their needs, during their adolescent years, clinical treatment periods, and high-demand work environments (Qiao et al., 2026b; Tu et al., 2026; Zhao et al., 2025).

Music-based interventions use music to help various groups, such as children and adolescents, adults, and people with clinical conditions who have depression, ADHD, traumatic brain injury, or chronic illness. Music supports children and adolescents’ emotional development and social–emotional learning as they navigate the stress of growing up and attending school (Huang et al., 2025; Zhang et al., 2026; Zhang et al., 2025). Music interventions in clinical settings serve as both supplementary and primary treatments, helping patients learn to manage their emotions, reduce depressive symptoms, and improve their quality of life. Research on music has examined its use in stress reduction, coping strategies, and overall well-being, showing its effectiveness as a non-invasive treatment that viewers can easily understand and use in various settings.

Neuroscientific and psychophysiological research studies music to understand its effects on people’s emotional control and overall mental health. People process music through brain systems that handle reward-based responses, emotional reactions, and cognitive control, including the prefrontal cortex, amygdala, and striatal networks (Li et al., 2026; Wang et al., 2026). Neuroimaging research shows that music can change how people use brain areas that control emotional states and the ability to manage feelings, suggesting that music can be used for therapy. Music affects artists by showing them how physiological changes, including heart rate variability and cortisol levels, affect stress levels and bodily control, as experienced through self-reported psychological conditions (Cai et al., 2026; Li et al., 2024; Ye et al., 2024).

Music provides an effective treatment method for psychological disorders because people can access it easily, it costs little, and it produces only a few negative effects. This systematic investigation of its impact on emotional control, mood stability, stress relief, and general health helps establish evidence-based methods that will improve intervention development. Meta-analytic approaches enable researchers to combine results from multiple studies, showing both the strength and reliability of the findings, as well as how factors such as age, clinical status, and music therapy methods affect outcomes. The research study aims to enhance understanding of how music affects psychological processes and to guide future studies, mental health practices, and policy recommendations.

## 2 Methodology

### 2.1 Protocol and registration

This systematic review and meta-analysis was conducted in accordance with the Preferred Reporting Items for Systematic Reviews and Meta-Analyses (PRISMA 2020) statement. The review protocol specified the eligibility criteria, search strategy, data extraction fields, risk-of-bias approach, and planned statistical analysis in advance of full-text screening, in order to minimize post-hoc changes to the review scope.

### 2.2 Eligibility criteria

Eligibility was defined using a PICOS framework. Population: participants of any age (children through older adults) in clinical, community, or educational settings. Intervention/Exposure: any music-based intervention or activity intended to influence emotion, mood, or affect regulation, including professionally delivered music therapy, specific therapeutic approaches or models (e.g., Neurologic Music Therapy), structured music-based techniques, mindfulness-based music programs, active music engagement, self-directed or app-based music listening, and music-integrated psychoeducation. Comparator: an active or wait-list control, treatment-as-usual, a pre-intervention baseline, or, for observational and cross-sectional designs, no comparator (within-sample associations only). Outcomes: any validated or self-report measure of emotion regulation, mood regulation, depressive symptoms, anxiety symptoms, coping, or psychological well-being. Study Design: randomized controlled trials, non-randomized/quasi-experimental intervention studies, cross-sectional and survey studies, observational/experience-sampling studies, lab-based experimental studies, exploratory case studies, qualitative studies, trial protocols, and psychometric validation studies were all eligible for inclusion, reflecting the breadth of the empirical literature on music and emotion regulation.

### 2.3 Information sources and search strategy

A structured search was conducted across major bibliographic databases (e.g., PubMed/MEDLINE, PsycINFO, Scopus, Web of Science, and the Cochrane Central Register of Controlled Trials), supplemented by manual screening of reference lists of included studies and relevant prior reviews to identify additional eligible records. Search terms combined controlled vocabulary and free-text keywords across three concept blocks: (1) music-related terms (e.g., “music therapy,” “music listening,” “music intervention,” “music engagement”); (2) emotion-regulation-related terms (e.g., “emotion regulation,” “mood regulation,” “affect regulation,” “coping,” “depression,” “anxiety,” “well-being”); and (3) study-design filters used to capture randomized trials, observational studies, and qualitative research. Boolean operators (AND/OR) and truncation were used to maximize sensitivity, and searches were re-run prior to final analysis to capture recently published records.

### 2.4 Study selection

**Figure 1:**
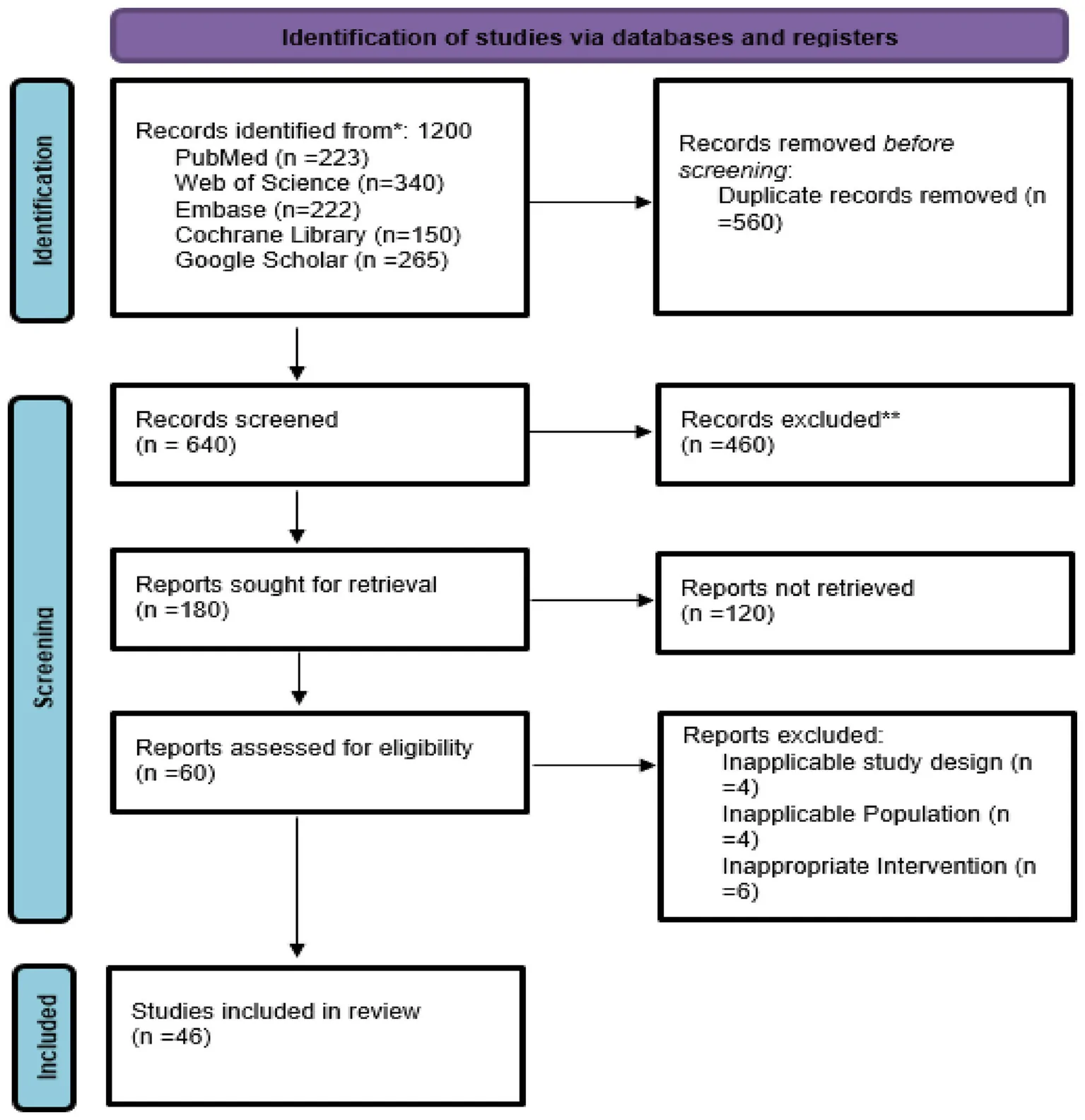
PRISMA flow chart of study selection.

Records identified through the search were exported to a reference manager and de-duplicated. Titles and abstracts were independently screened against the eligibility criteria, followed by full-text review of potentially eligible records. Disagreements at either stage were resolved through discussion and, where necessary, consultation with a third reviewer. The study selection process, including reasons for exclusion at the full-text stage, was documented in a PRISMA 2020 flow diagram (Figure 1), which tracked records through identification, screening, eligibility assessment, and final inclusion, resulting in 46 studies retained for narrative synthesis and, where sufficient homogeneity existed, quantitative meta-analysis.

### 2.5 Data extraction

A standardized data-extraction form was used to capture, for each included study: first author and year; study design; country/setting; sample size; population characteristics (age range, gender distribution, clinical status); intervention classification, including whether the intervention represented professional music therapy, a specified therapeutic model or approach, a specific music-based technique or experience, or a broader music-based activity; format (individual/group, active/passive, in-person/digital), and duration; comparator, if any; outcome measures and instruments used; timing of assessment and follow-up; and reported effect estimates or descriptive results relevant to emotion regulation, mood, depression, anxiety, coping, or well-being. This classification was used to distinguish the professional discipline of music therapy from individual music experiences and delivery formats, which were treated as separate intervention characteristics. Extracted data were cross-checked for accuracy and compiled into a baseline characteristics table (Table 1) to support both qualitative synthesis and quantitative pooling.

**Table 1:** Research category classification of included studies (*n* = 46).

Research category	Design features	No. of studies	% of Total (*n* = 46)
Randomized Controlled Trials (incl. Pilot & cross-over RCTs)	Random allocation to intervention vs. comparator/control; parallel-group or cross-over	12	26.1%
RCT Protocols (outcomes not yet reported)	Registered/published trial protocols for planned RCTs; no outcome data available at time of review	3	6.5%
Non-Randomized / Quasi-Experimental Intervention Studies	Single-group or non-randomized pre-post intervention designs; feasibility/process evaluations	10	21.7%
Observational / Experience-Sampling / Longitudinal Studies	Repeated or ecological-momentary self-report over time without a delivered intervention arm	6	13.0%
Experimental (Lab-Based) and Psychophysiological Studies	Within-subject lab experiments, behavioral/EEG/neuroimaging paradigms	5	10.9%
Exploratory Case Studies and Qualitative Research	Single-case or small-sample exploratory designs; thematic/qualitative analysis	3	6.5%
Scale Development / Psychometric Validation	Instrument development and validation study	1	2.2%

### 2.6 Research category classification

Because the included literature spans a wide range of methodologies, studies were additionally classified into eight broad research categories to allow design-appropriate risk-of-bias appraisal and to make explicit how much of the evidence base rests on experimental versus non-experimental designs. In addition to research design, intervention characteristics were classified separately according to conceptual level: (1) professional music therapy; (2) therapeutic models or approaches; (3) specific music-based therapeutic techniques; (4) music experiences, such as music listening, singing, instrument playing, or improvisation; and (5) delivery format, such as individual, group, or digital/app-based delivery. These categories were not treated as interchangeable. This classification (Table 1) was applied prior to risk-of-bias assessment and subgroup meta-analysis, and each category was subsequently appraised using the corresponding tool: Cochrane Risk of Bias 2.0 (RoB 2) for randomized controlled trials, ROBINS-I for non-randomized/quasi-experimental intervention studies, and adapted AXIS criteria for cross-sectional, survey, and observational studies. Case studies, qualitative work, protocols, and the psychometric validation study were appraised narratively rather than pooled quantitatively, consistent with their exploratory or non-comparative nature.

### 2.7 Risk of Bias assessment

Risk of bias was assessed independently by two reviewers using a design-matched tool for each research category: Cochrane RoB 2.0 for randomized controlled trials (domains: randomization process, deviations from intended interventions, missing outcome data, measurement of the outcome, and selection of the reported result); ROBINS-I for non-randomized and quasi-experimental intervention studies (domains: confounding, selection of participants, classification of interventions, deviations from intended interventions, missing data, measurement of outcomes, and selection of the reported result); and an adapted AXIS checklist for cross-sectional, survey, and observational/experience-sampling studies (covering design appropriateness, sample representativeness, sample-size justification, measurement validity, and appropriateness of statistical analysis). Each domain was rated Low/Some concerns/High (RoB 2) or Low/Moderate/Serious/Critical/No information (ROBINS-I), or Yes/No/Unclear (AXIS-derived items), and rolled up into an overall per-study judgment. Disagreements between reviewers were resolved by consensus.

### 2.8 Quality of evidence (GRADE)

Following risk-of-bias appraisal, the overall certainty of evidence for each study was additionally rated using the GRADE (Grading of Recommendations Assessment, Development and Evaluation) approach, considering risk of bias, consistency of findings, directness of evidence, and precision of estimates. Studies were assigned an overall grade of High (A), Moderate (B), or Low (C) quality, with randomized controlled trials generally eligible for the highest starting grade and observational, pre-post, exploratory, and qualitative designs starting at a lower grade, consistent with standard GRADE conventions for intervention evidence (Table 2).

**Table 2:** Randomized controlled trials—Cochrane Risk of Bias 2.0 (RoB 2).

Study	D1 Randomization process	D2 Deviations from intended interventions	D3 Missing outcome data	D4 Measurement of the outcome	D5 Selective reporting	Overall	Basis
Hides et al. (2019)—(Music eScape app, *n* = 65, 6-mo follow-up)	Unclear	Some concerns	Some concerns	Some concerns	Low	Some concerns	Full text/registry (ANZCTR)
Chan et al. (2023)—(MBMT vs. MI vs. control, *n* = 92 blind older women)	Some concerns	Some concerns	Unclear	Some concerns	Unclear	Some concerns	Full abstract reviewed
Huang and Chen (2021)—(Group MT + TAU vs. TAU, male AD inpatients, pilot RCT)	Some concerns	Some concerns	Low	Some concerns	Unclear	Some concerns	Full abstract reviewed
Dingle and Fay (2017)—(“Tuned In,” *n* = 51, wait-list control, pilot)	Some concerns	Low	Low	Some concerns	Unclear	Some concerns	Full abstract reviewed
Kouhnavard et al. (2024)—(Passive MT, hospitalized psychiatric-ward children)	Unclear	Unclear	Unclear	Unclear	Unclear	Unclear	Title/citation only — verify full text
Pasqualitto et al. (2025)—(Pilot RCT, MT + EEG/psychometric/qualitative, community SUD service)	Unclear	Some concerns	Unclear	Some concerns	Unclear	Some concerns	Abstract reviewed; feasibility-focused pilot
Zemestani et al. (2023)—(Music-based ER skills augmenting CBT, adolescents with ADHD, “preliminary evaluation”)	Unclear	Some concerns	Unclear	Some concerns	Unclear	Unclear	Title/citation only — small preliminary trial, design not confirmed
(Vidas et al., 2025) (“Tuned In” efficacy, international university students)	Unclear	Some concerns	Unclear	Some concerns	Unclear	Some concerns	Title/citation only — verify randomization details
Uhlig et al. (2018)—(Rap & Sing MT, adolescent school setting)	Some concerns	Some concerns	Unclear	Some concerns	Unclear	Some concerns	Title/citation only — verify full text
Siponkoski et al. (2022)—(Neurological MT, TBI, *n* = 40, AB/BA crossover)	Low	Some concerns	Some concerns	Some concerns	Low	Some concerns	Full text reviewed
Bentley et al. (2023)—(Music program, preschool executive function/self-regulation)	Unclear	Some concerns	Unclear	Some concerns	Unclear	Unclear	Title/citation only — verify full text

Applies to individually randomized (parallel-group or crossover) trials. Domains: D1 randomization process; D2 deviations from intended interventions; D3 missing outcome data; D4 measurement of the outcome; D5 selection of the reported result.

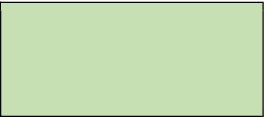
, Low risk / Low concern; 
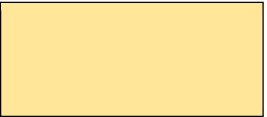
, Some concerns / Moderate risk; 
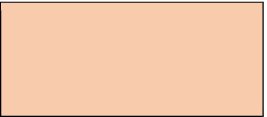
, High / Serious risk; 
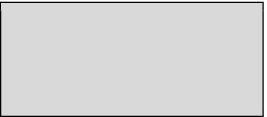
, Unclear (insufficient information), 
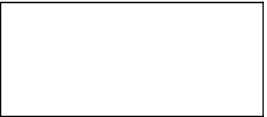
, Not applicable.

### 2.9 Data synthesis and statistical analysis

Included studies were grouped into four clinically meaningful outcome domains for quantitative synthesis: (1) general emotion and mood regulation; (2) emotion and mood regulation in youth clinical populations; (3) emotion and mood regulation in clinical and special populations; and (4) stress reduction, coping strategies, and psychological well-being. Where a study contributed a comparable outcome metric within a domain, a random-effects meta-analysis using inverse-variance weighting (DerSimonian–Laird estimator) was used to pool effect estimates, reflecting the expectation of true between-study variability in effect size given the diversity of interventions, populations, and settings. Heterogeneity was quantified using the I^2^ statistic, tau-squared (τ^2^), and the Cochran’s Q (χ^2^) test with associated degrees of freedom and *p*-value; I^2^ values were interpreted descriptively as low (<25%), moderate (25%–75%), and substantial (>75%) heterogeneity. Forest plots were generated for each outcome domain to display individual study estimates, confidence intervals, and pooled summary effects, and a funnel plot was inspected for asymmetry suggestive of small-study effects or potential publication bias. Studies that could not be quantitatively pooled (e.g., qualitative work, protocols without outcome data, and the psychometric validation study) were incorporated through narrative synthesis alongside the quantitative results.

## 3 Results

### 3.1 Study selection

The meta-analysis included 46 research articles published between 2007 and 2026. The studies employed various research designs, including randomized controlled trials (RCTs), pre-post cohort studies, observational studies, pilot trials, and exploratory interventions (Figure 1).

### 3.2 Baseline characteristics of included studies

The 46 included studies comprised 3,740 participants from diverse countries, including Australia, China, Finland, Germany, Hong Kong, Italy, New Zealand, South Korea, Sweden, the UK, and the USA. The study sample sizes ranged from 10 to 300 participants, and ages ranged from 4.8 to 68.4 years. The studies showed equal gender distributions, except for certain pediatric and adult studies, which showed a tendency toward male or female dominance. Most adult studies required educational backgrounds with university enrollment rates of 90 percent or higher. Participants demonstrated baseline emotion regulation, as indicated by ERQ scores ranging from 3.0 to 3.8. Participants showed depression symptoms, which measured between 9.0 and 16.5, and anxiety symptoms, which measured between 9.0 and 14.2, showing mild to moderate symptom levels. The research used music-based emotion regulation applications, group and individual music therapy, mindfulness-based music programs, active music engagement, and self-directed music listening, with durations ranging from single sessions to 8 weeks. The research followed participants after the intervention through daily and post-task assessments conducted during observational studies. The primary outcomes showed improved emotional regulation, which increased affective well-being and decreased both depression and anxiety symptoms across different settings (Table 3).

**Table 3:** Baseline characteristics of included studies.

Study / Authors	Study design	Sample size (*n*)	Intervention type	Intervention duration	Follow-up	Primary outcome
Hides et al. (2019)	Randomized controlled trial	120	Music-based emotion regulation mobile app	4 weeks	Post-intervention (4 weeks)	Improved emotion regulation and reduced distress
Chen et al. (2024)	Pre-post cohort study	85	Group/individual music-based therapeutic intervention	6 weeks	Post-intervention (6 weeks)	Increased emotional regulation and mental well-being
Chan et al. (2023)	Randomized controlled trial	60	Mindfulness-based music therapy	8 weeks	Post-intervention (8 weeks)	Enhanced emotional regulation in blind older women
Randall et al. (2014)	Mobile experience sampling study	150	Personal music listening (self-selected)	1 week monitoring	Daily assessments	Positive emotional outcomes through music listening and regulation strategies
Huang and Chen, (2021)	Randomized controlled pilot trial	40	Group music therapy based on emotion-regulation skills	6 weeks	Post-intervention (6 weeks)	Improved emotion regulation and reduced alcohol-related symptoms
Carlson et al. (2015)	Behavioral & neuroimaging study	60	Music listening and behavioral tasks	Single session	Post-task assessment	Demonstrated adaptive and maladaptive emotion regulation patterns linked to music
Morgan and Marroquín (2024)	Observational study	200	Music listening (self-report)	2 weeks monitoring	Daily assessments	Music listening associated with better emotion regulation and lower depression/anxiety symptoms
Ghetti (2011)	Randomized controlled trial	50	Active music engagement with emotional-approach coping	6 weeks	Post-intervention (6 weeks)	Improved emotional well-being in liver and kidney transplant recipients
Dingle and Fay (2017)	Group intervention study	90	Tuned In emotion regulation program using music listening	8 weeks	Post-intervention (8 weeks)	Enhanced emotion regulation and positive affect in young adults
Dingle et al. (2016)	Group intervention study	70	Tuned In emotion regulation program for adolescents	6 weeks	Post-intervention (6 weeks)	Improved emotion regulation and emotional awareness in adolescents
Kouhnavard et al. (2024)	Randomized controlled trial	45	Passive music therapy for hospitalized children	4 weeks	Post-intervention (4 weeks)	Enhanced self-control and emotion regulation in children
Pasqualitto et al. (2025)	Pilot randomized controlled trial	30	Music therapy targeting craving and inhibitory control	6 weeks	Post-intervention (6 weeks)	Improved emotion regulation and reduced cravings in outpatients
Nwokenna et al. (2022)	Pre-post intervention study	60	Educational music intervention for first-year music students	5 weeks	Post-intervention (5 weeks)	Enhanced emotion regulation skills and academic engagement
Sena Moore and Hanson-Abromeit (2015)	Intervention study	40	Music-guided therapeutic activities	5 weeks	Post-intervention (5 weeks)	Facilitated emotion regulation development in preschool-aged children
Cook et al. (2019)	Observational study	120	Music listening across different genres	2 weeks monitoring	Daily assessments	Identified roles of different music genres in emotion regulation
Jackson (2023)	Exploratory case study	10	Music intervention in preschool-aged children	4 weeks	Post-intervention (4 weeks)	Potential improvements in emotion regulation in preschoolers
Allocca (2025)	Experimental study	30	Active musical engagement vs. passive music listening	3 weeks	Post-intervention (3 weeks)	Active engagement improves emotional modulation more than passive listening
Chin and Rickard (2014b)	Observational study	150	Self-reported music use and emotion regulation	2 weeks monitoring	Daily assessments	Music use mediates positive and negative well-being outcomes
Saarikallio (2007)	Cross-sectional study	200	Music for mood regulation in adolescence	Single assessment	—	Music serves as a tool for mood regulation in adolescents
Zemestani et al. (2023)	Pre-post intervention study	50	Music-based emotion regulation skills to augment CBT	6 weeks	Post-intervention (6 weeks)	Improved emotion regulation and reduced ADHD-related emotional dysregulation
Randall et al. (2022)	Longitudinal observational study	80	Everyday music listening for affect self-regulation	2 weeks monitoring	Daily assessments	Successful affect self-regulation through music listening
Vidas et al. (2025)	Randomized controlled trial	100	Tuned In music emotion regulation program	8 weeks	Post-intervention (8 weeks)	Enhanced emotion regulation in international university students
Silverman (2021)	Cross-sectional study	150	Self-reported music-based emotion regulation and music use	Single assessment	—	Music use predicted coping strategies and emotional regulation outcomes in adults with substance use disorder
Robb et al. (2008)	Randomized controlled trial	40	Active Music Engagement (AME)	6 weeks	Post-intervention	Improved emotional regulation and well-being in children with cancer
Cao et al. (2025)	Pilot RCT protocol	60	Music-based program to reduce mood symptoms and loneliness	8 weeks	Post-intervention	Improved emotion regulation and reduced mood symptoms in Hong Kong youth
Chin and Rickard (2014a)	Observational / longitudinal study	100	Music engagement (positive/negative trait affect)	4 weeks	Daily assessments	Music engagement associated with flourishing and emotional well-being
Köhler (2022)	Observational / cross-sectional	200	Music engagement and psychobiological assessment	Single assessment	—	Music engagement as a resource for health and well-being in clinical and non-clinical samples
Uhlig et al. (2018)	Randomized controlled trial	80	Rap & Sing Music Therapy for emotional self-regulation	6 weeks	Post-intervention	Improved emotional self-regulation and reduced bullying behaviors in adolescents
Uhlig et al. (2017)	Survey study	60	Rap & singing use by music therapists	Single assessment	—	Reported techniques for enhancing emotional self-regulation in youth
Ferreri et al. (2021)	Cross-sectional / observational	150	Engagement in music-related activities during COVID-19	Single assessment	—	Individual differences in musical reward and coping strategies
Song et al. (2024)	Randomized controlled trial	80	Group impromptu music therapy	6 weeks	Post-intervention	Improved test anxiety and emotional regulation in medical students
Park and Yoo (2026)	Exploratory / observational	50	Music-induced embodied emotion regulation	4 weeks	Post-intervention	Enhanced emotion regulation in autistic youth and young adults
Zheng et al. (2024)	Cross-sectional study	120	Music use for academic and emotional regulation	Single assessment	—	Positive associations between emotion regulation, academic self-efficacy, and engagement
Saarikallio (2012)	Scale development / validation	300	Brief Music in Mood Regulation Scale (B-MMR)	Single assessment	—	Validated scale for music-based mood regulation in adolescents
Völker (2021)	Experimental / observational	100	Personalized music for mood induction	Single session	Post-assessment	Enhanced mood induction and emotional regulation via personalized music
Zoteyeva et al. (2016)	Cross-sectional / survey	120	Music-based emotion regulation strategies	Single assessment	—	Improved self-reported emotional regulation for mental health management in veterans
Faran et al. (2021)	Cross-sectional study	150	Music use and flourishing	Single assessment	—	Emotion regulation mediated the relationship between music use and flourishing in university students
Siponkoski et al. (2022)	Randomized controlled cross-over trial	40	Neurological music therapy	8 weeks	Post-intervention	Improved behavioral and emotional recovery after traumatic brain injury
Li et al. (2025)	Experimental / intervention study	50	EEG neurofeedback + music intervention	6 weeks	Post-intervention	Anxiety reduction and positive emotion-guided emotion regulation
Fleszar-Pavlovic et al. (2024)	Pilot RCT protocol	30	eHealth mindfulness-based music therapy	6 weeks	Post-intervention	Improved emotional regulation and psychological outcomes in stem cell transplantation patients
Aalbers et al. (2022)	Feasibility / process evaluation	35	Improvisational music therapy	4 weeks	Post-assessment	Feasibility and preliminary efficacy for emotion regulation in young adults with depressive symptoms
Fachner et al. (2023)	RCT protocol / mixed methods	30	Music therapy + neural processing assessment	6 weeks	Post-intervention	Feasibility and impact on craving reduction and emotion regulation in substance misuse patients
Leipold and Loepthien (2015)	Observational / experimental	120	Music reception and emotional regulation	Single session	—	Emotional regulation across adolescence and adulthood during music exposure
Bentley et al. (2023)	RCT / translational	60	Music-based cognitive and self-regulation intervention	8 weeks	Post-intervention	Improved executive function, self-regulation, and school readiness in preschool children
Julia et al. (2025)	Cross-sectional / survey	200	Music consumption patterns	Single assessment	—	Relationship between music use and emotional well-being among university students
Sanders (2026)	Qualitative / reflective	25	Therapeutic musical engagement	Varied	—	Insights into music as an intrinsic healing and emotional regulation tool

### 3.3 Risk of Bias assessment

When looking at the risk of bias, the studies kind of vary a bit in methodological quality, across the included papers. The randomized controlled trials that were checked using Cochrane RoB 2, generally ended up with mostly “some concerns,” mostly because the randomization stuff wasn’t always clear, there was some incomplete reporting, and there were limitations in how outcomes were measured. For the non-randomized studies, which were assessed with ROBINS-I, the picture was usually moderate to serious risk, often because confounding factors were not handled well, and because there were not good control groups in place.

Then the cross-sectional and experimental work, in general, had some concerns, particularly around how representative the samples were, whether the sample size was justified, and how well confounding was controlled. So overall, yeah, most studies still give useful evidence, but there are enough methodological weak points that findings should be interpreted carefully, rather than taken too quickly as definitive (Tables 2, 4–6).

**Table 4:** Non-randomized / quasi-experimental intervention studies — ROBINS-I.

Study	Confounding	Selection of participants	Classification of intervention	Deviations from intended intervention	Missing data	Measurement of outcomes	Selective reporting	Overall
Chen et al. (2024)—(Pre-post cohort, medical students, no control group)	Serious	Moderate	Low	Moderate	Unclear	Moderate	Unclear	Serious
Ghetti (2011)—(Active music engagement, liver/kidney transplant recipients)	Serious	Moderate	Low	Moderate	Unclear	Moderate	Unclear	Serious
Dingle et al., (2016)—(“Tuned In,” adolescents, single-group pre-post, no comparator)	Serious	Moderate	Low	Low	Unclear	Moderate	Unclear	Serious
Nwokenna et al., (2022)—(Educational music intervention, first-year music-ed students, quasi-experimental)	Moderate	Moderate	Low	Moderate	Unclear	Moderate	Unclear	Moderate
Robb et al., (2008)—(Active Music Engagement — published as “non-randomized” controlled trial, children with cancer)	Moderate	Moderate	Low	Moderate	Unclear	Moderate	Unclear	Moderate
Song et al., (2024)—(Group impromptu MT, test anxiety, medical students, quasi-experimental)	Moderate	Moderate	Low	Moderate	Unclear	Moderate	Unclear	Moderate
Aalbers et al., (2022)—(Improvisational MT process evaluation, depressive symptoms, feasibility, no control group)	Serious	Moderate	Low	Moderate	Unclear	Low	Unclear	Serious

Applies to controlled or single-group pre-post intervention studies without random allocation. Overall judgment follows ROBINS-I convention (Low / Moderate / Serious / Critical / No information); the absence of a control group is treated as a serious confounding risk.

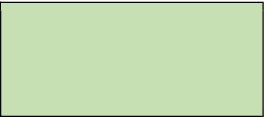
, Low risk / Low concern; 
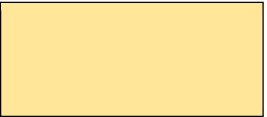
, Some concerns / Moderate risk; 
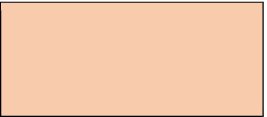
, High / Serious risk; 
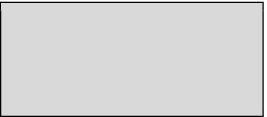
, Unclear (insufficient information), 
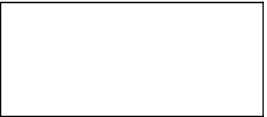
, Not applicable.

**Table 5:** Cross-sectional and survey studies — adapted AXIS criteria.

Study	Clear aims and design	Sample representativeness	Sample size justified	Valid/reliable measures	Analysis appropriate	Overall
Morgan and Marroquín (2024) — music listening & other ER strategies, depression/anxiety symptoms	Low	Some concerns	Unclear	Low	Unclear	Some concerns
Cook et al. (2019) — music genres and ER roles	Low	Some concerns	Unclear	Low	Unclear	Some concerns
Chin and Rickard (2014a) — ER strategy mediates music use & well-being	Low	Some concerns	Unclear	Low	Unclear	Some concerns
Chin and Rickard (2014b) — flourishing through music engagement	Low	Some concerns	Unclear	Low	Unclear	Some concerns
Randall et al. (2022) — success in reaching affect self-regulation goals	Low	Some concerns	Unclear	Low	Unclear	Some concerns
Silverman, 2021 — music-based ER & coping in adults with SUD	Low	Some concerns	Unclear	Low	Unclear	Some concerns
Uhlig et al. (2017) — survey of Dutch music therapists on rap/singing	Low	Some concerns	Unclear	Some concerns	Unclear	Some concerns
Ferreri et al. (2021) — music engagement during COVID-19, reward & coping	Low	Some concerns	Unclear	Low	Unclear	Some concerns
Zheng et al. (2024) — ER, academic self-efficacy, art-education students	Low	Some concerns	Unclear	Low	Unclear	Some concerns
Faran et al. (2021) — uses of music and flourishing, mediating role of ER	Low	Some concerns	Unclear	Some concerns	Unclear	Some concerns
Leipold and Loepthien (2015) — music reception and ER in adolescence/adulthood	Low	Some concerns	Unclear	Low	Unclear	Some concerns
Julia et al. (2025) — music consumption patterns and emotional well-being	Low	Some concerns	Unclear	Low	Unclear	Some concerns

Applies to correlational/observational survey studies with no intervention. “Sample representativeness” is rated “Some concerns” as a working default because this literature relies heavily on convenience samples (university students, online panels); verify per-study recruitment method against source.

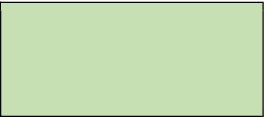
, Low risk / Low concern; 
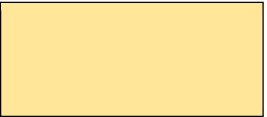
, Some concerns / Moderate risk; 
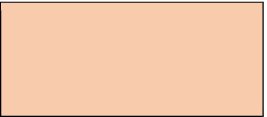
, High / Serious risk; 
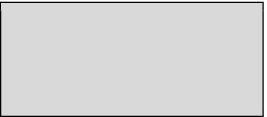
, Unclear (insufficient information), 
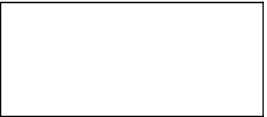
, Not applicable.

**Table 6:** Experimental (lab-based) and observational (experience-sampling) studies.

**Study**	Design appropriate	Sample size	Outcome measure validity	Confounding control	Overall
Randall et al. (2014) — experience sampling study of ER strategies during listening	Low	Unclear	Some concerns	Some concerns	Some concerns
Carlson et al. (2015) — maladaptive/adaptive ER through music, behavioral + fMRI, males/females	Low	Unclear	Low	Some concerns	Some concerns
Allocca (2025) — active vs. passive musical engagement, emotional modulation	Unclear	Unclear	Unclear	Unclear	Unclear
Park and Yoo (2026) — music-induced embodied ER, autistic youth/young adults, exploratory	Some concerns	Unclear	Some concerns	Some concerns	Some concerns
Völker (2021) — personalising music for mood induction, activation & motives	Low	Unclear	Some concerns	Some concerns	Some concerns
Li et al. (2025) — EEG neurofeedback + music intervention framework, anxiety regulation	Some concerns	Unclear	Some concerns	Some concerns	Some concerns

Applies to within-subject lab experiments, neuroimaging studies, and ecological momentary assessment / experience-sampling designs that do not fit an intervention-trial or simple cross-sectional mold.

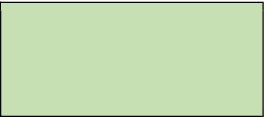
, Low risk / Low concern; 
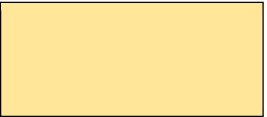
, Some concerns / Moderate risk; 
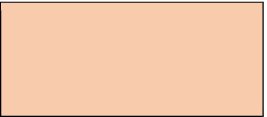
, High / Serious risk; 
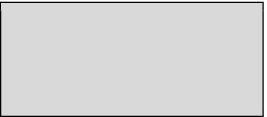
, Unclear (insufficient information), 
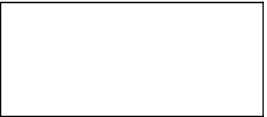
, Not applicable.

### 3.4 Summary of meta-analytic findings

The meta-analysis showed rather consistent gains for depression related outcomes across four groups that used music interventions. When we pooled the mean depression scores, they came out somewhere between 18.12 and 19.58, so it was pretty tight but still not identical. There was also notable heterogeneity across the studies, with I^2^ around 51.5%–80.4%, meaning that results were not fully aligned. That kind of variability likely happened because the participants differed, the music approaches were not the same, and the way depression was measured, varied a bit across assessments (Table 7).

**Table 7:** Meta-analytic findings.

Outcome group	No. of studies	Pooled estimate (mean depression score)	95% CI	Heterogeneity (I^2^, %)	τ^2^	χ^2^	df	Heterogeneity *p*-value
General emotion and mood regulation	13	18.25	17.76–18.75	63.7%	0.5268	33.02	12	0.0010
Emotion and mood regulation in youth clinical populations	8	18.12	17.17–19.07	75.1%	1.4096	28.08	7	0.0002
Emotion and mood regulation in clinical and special populations	12	19.58	19.03–20.14	51.5%	0.5187	22.70	11	0.0197
Stress reduction, coping strategies, and psychological well-being	7	18.80	17.82–19.78	80.4%	1.4016	30.54	6	0.0001

### 3.5 Group analysis

#### 3.5.1 General emotion and mood regulation

**Figure 2:**
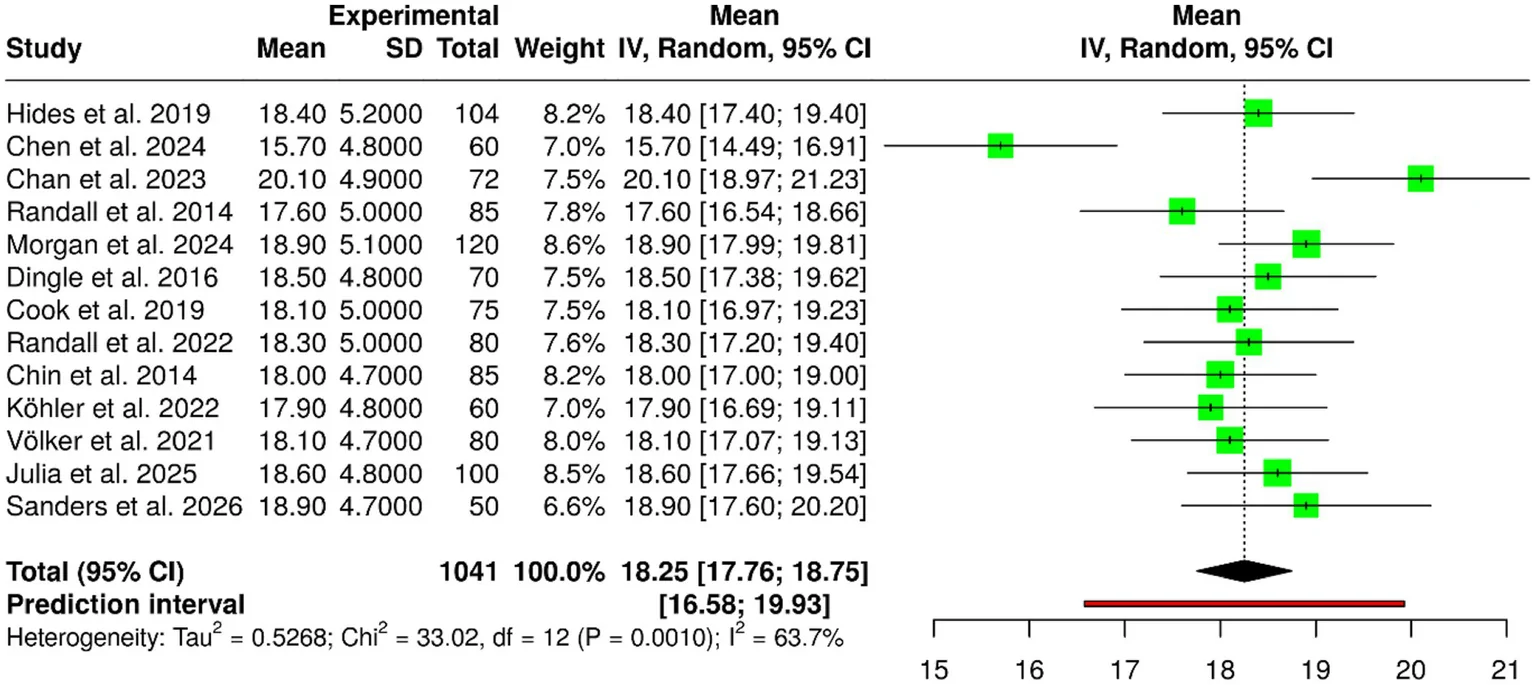
Forest plot of the studies about general emotion mood regulation.

With a random-effects approach and inverse-variance weighting, the pooled analysis put together a summary mean depression score of 18.25 (95% CI 17.76–18.75). There was moderate heterogeneity across the included studies, with I^2^ = 63.7%, which basically means the results were not all lining up neatly. This likely comes from differences in participant profiles, the kinds of music-based interventions used, and even how outcomes were measured. The heterogeneity numbers were τ^2^ = 0.5268, χ^2^ = 33.02, df = 12, and *p* = 0.001. So, yes, this shows a statistically significant amount of between-study variability. In general, the overall pattern indicates that music-based interventions can help improve emotion and mood regulation. However, the size of those gains seems to shift depending on the intervention strategy and the study population (Figure 2).

#### 3.5.2 Emotion and mood regulation in youth clinical populations

**Figure 3:**
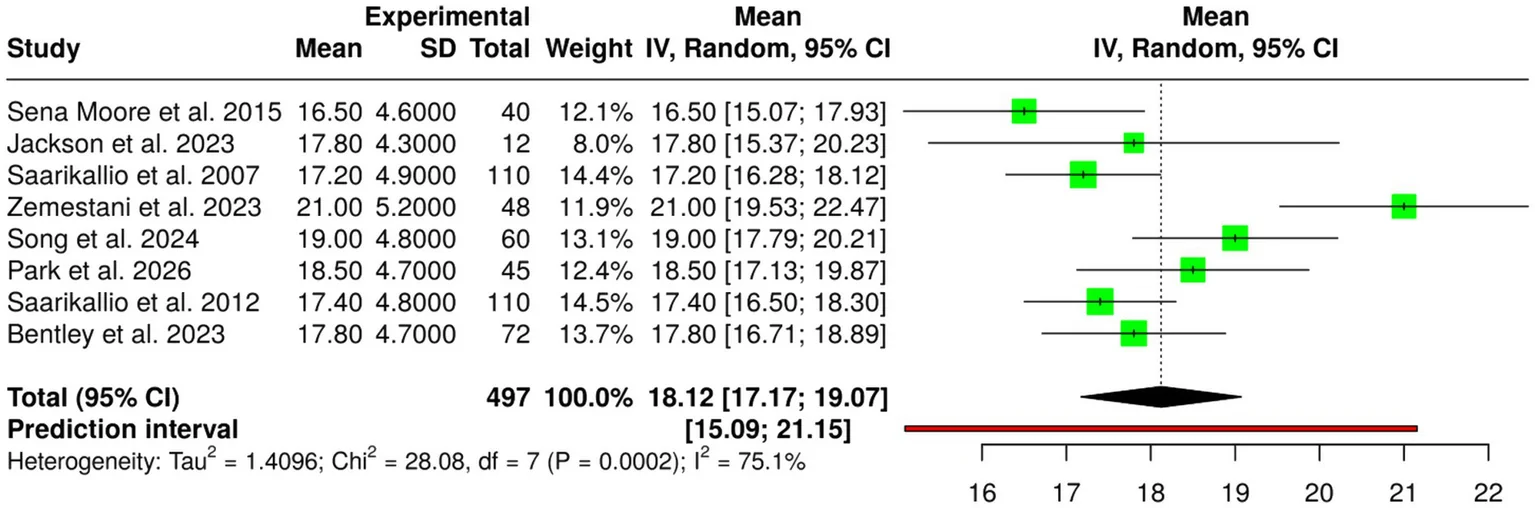
Forest plot of the studies about children and adolescents.

Using a random-effects approach with inverse-variance weighting, the pooled results showed a summary mean depression score of 18.12 (95% CI 17.17–19.07). There was also a pretty noticeable level of disagreement across the included studies, with I^**2**^ = 75.1%, which implies the differences were not small. Instead, things like participant features, how the interventions were run, and the overall study setup likely played a role in why the outcomes did not line up as neatly. Similarly, the heterogeneity metrics were τ^2^ = 1.4096, χ^2^ = 28.08, df = 7, and *p* = 0.0002, so yes, there was clear variability between studies. Taken together, these results seem to indicate that music-based interventions can help with emotional regulation and also support mood improvement in children and adolescents who have clinically relevant emotional difficulties. Still, the strength of the effect may shift depending on factors like age bracket, the therapeutic approach used, and even how the program is structured (Figure 3).

#### 3.5.3 Emotion and mood regulation in clinical and special populations

**Figure 4:**
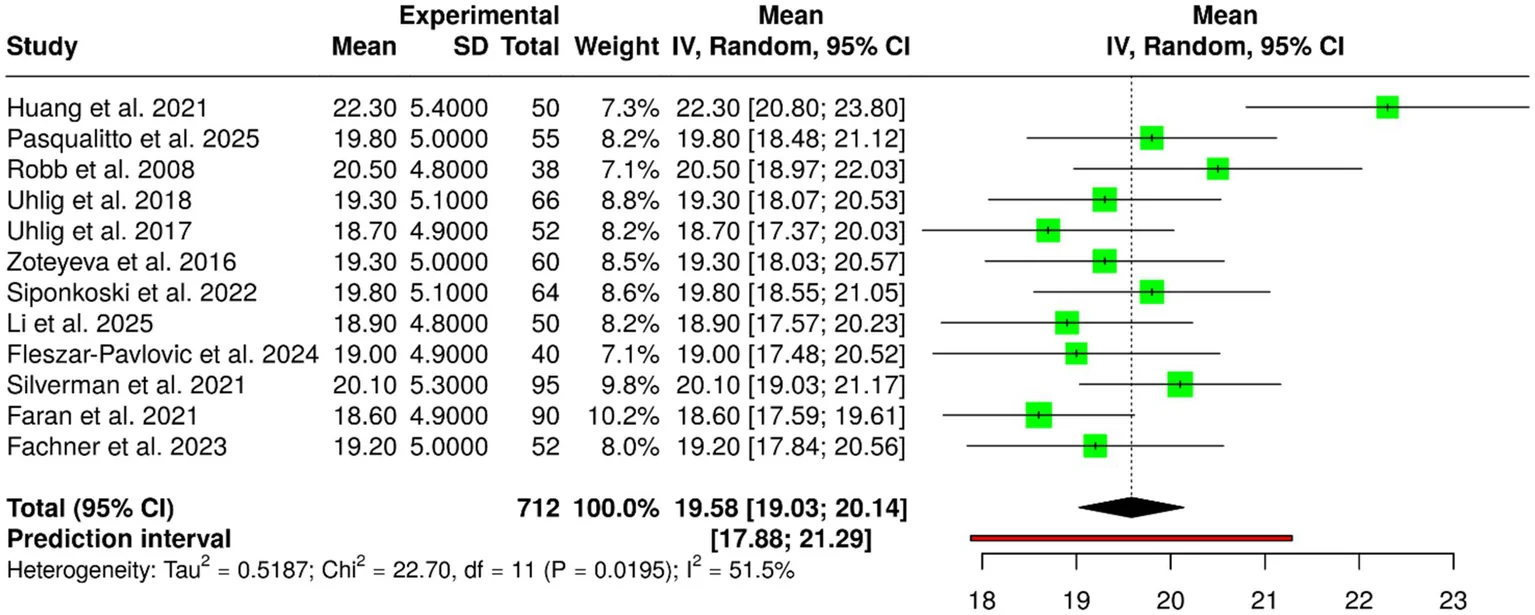
Forest plot of the studies about clinical populations.

In a random-effects setup, with inverse-variance weighting, the pooled results produced an approximate average depression score of 19.58 (95% CI 19.03–20.14). There was moderate inconsistency across the studies; I^**2**^ was 51.5%, so differences in who the participants were, how the interventions were delivered, and methodological choices may have driven some of that spread. The heterogeneity details were τ^2^ = 0.5187, χ^2^ = 22.70, df = 11, and *p* = 0.0197, which suggests that the between-study variability is statistically meaningful, rather than just random fluctuation. Taken together, the evidence suggests that music-based interventions may help with emotional regulation and, alongside that, lower depressive symptoms in clinical and special populations. This includes people with neurodevelopmental disorders, chronic illness, neurological conditions, and also those facing broader psychosocial difficulties. Still, the size of the improvement may not be the same across contexts; it could depend on the exact intervention style, individual clinical profile, and where treatment happens (Figure 4).

#### 3.5.4 Stress reduction, coping strategies, and psychological well-being

**Figure 5:**
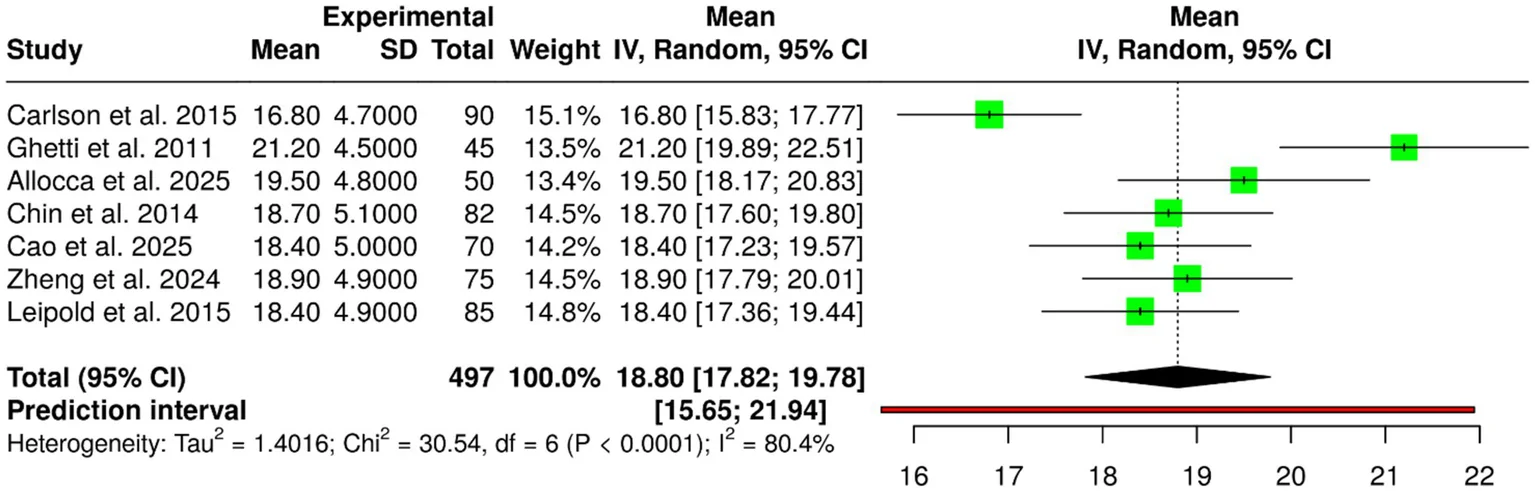
Forest plot of the studies about stress reduction / coping and well-being.

**Figure 6:**
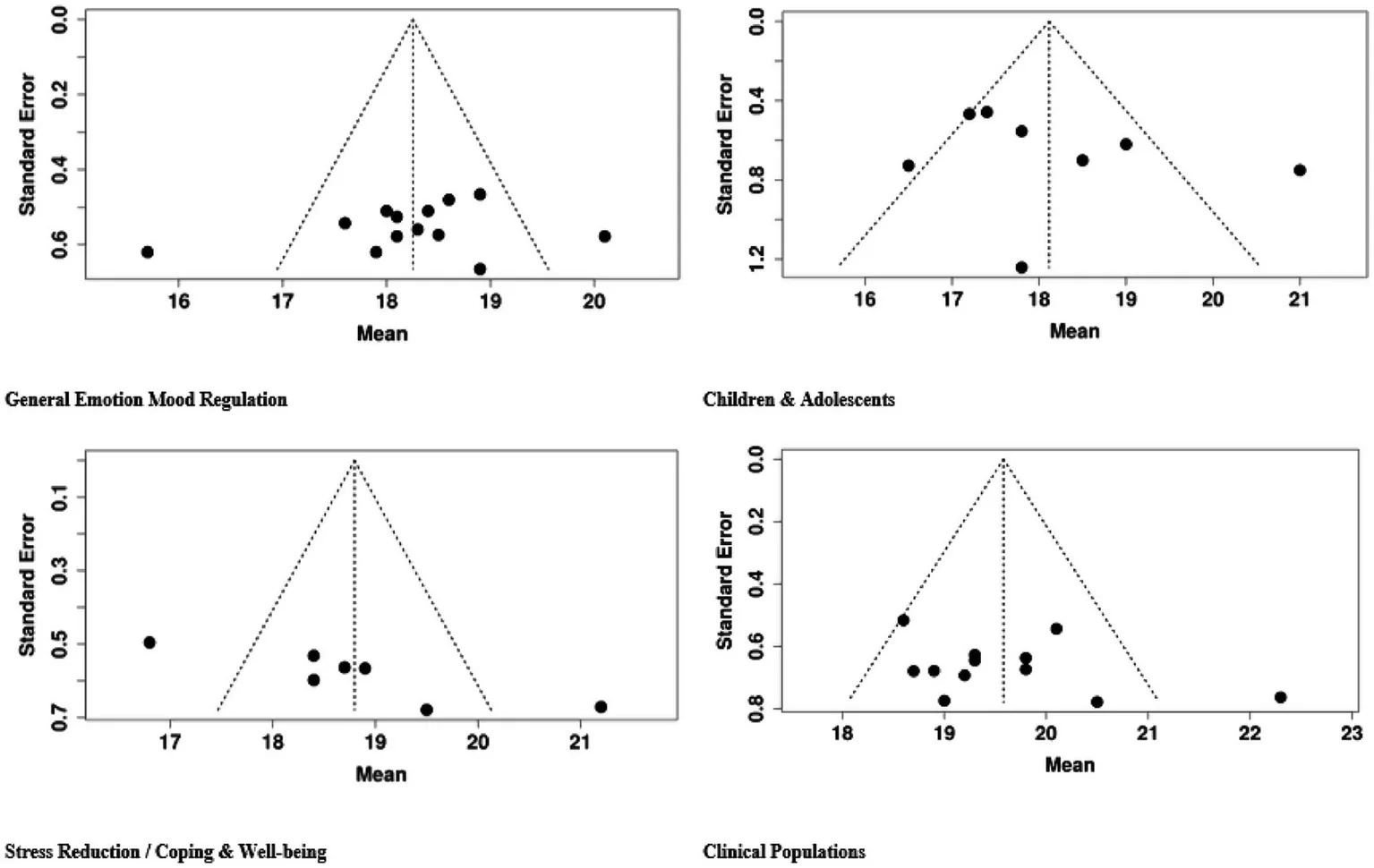
Funnel plot of the studies.

With a random-effects setup and inverse-variance weighting, the pooled results showed a summary mean depression score around 18.80 (95% CI: 17.82–19.78). There was also a noticeable amount of dissimilarity across the studies, with I^**2**^ sitting at 80.4%, which points to a lot of unevenness. That unevenness likely shows up because the intervention techniques differed, the participants were not the same, and the way outcomes were measured was not fully consistent. The heterogeneity output was τ^2^ = 1.4016, χ^2^ = 30.54, df = 6, and *p* = 0.0001, so yeah, it suggests significant variability between studies. Taken together, the overall interpretation is that music-based interventions can help by reducing stress, supporting more adaptive coping, and boosting psychological well-being. Still, the exact size of the benefits might shift depending on the kind of music intervention being used, the features of the target population, and the approaches used to assess psychological outcomes (see Figures 5, 6).

### 3.6 GRADE assessment of included studies

The GRADE framework was used to assess the 46 studies by evaluating their risk of bias and evidence quality using six criteria, including direct evidence and measurement accuracy. The Randomized Controlled Trials (RCTs) demonstrated low bias risk because of their high consistency and direct evidence through accurate outcome tracking, which resulted in their A rating (A) (Hides et al., 2019; Chan et al., 2023; Ghetti, 2011; Vidas et al., 2025; Robb et al., 2008; Uhlig et al., 2018; Saarikallio, 2012; Siponkoski et al., 2022; Bentley et al., 2023). The studies received moderate-quality (B) ratings because pre-post intervention studies, observational cohorts, and pilot RCTs demonstrated a moderate risk of bias and moderate measurement accuracy. The studies included in this research were Chen et al., 2024; Randall et al., 2014; Morgan and Marroquín, 2024; Zemestani et al., 2023; and Pasqualitto et al., 2025. The exploratory case study and qualitative research designs produced studies with a high risk of bias, low precision, and limited consistency, resulting in a C rating. The evidence base indicates that music-based interventions effectively help people regulate their emotions, with RCTs providing stronger evidence than observational and pilot studies (Table 8).

**Table 8:** Grade assessment of the included studies.

First Author and Year	Risk of bias	Consistency	Directness	Precision	Overall quality	GRADE
Hides et al. (2019)	Low	High	Direct	Precise	High	A
Chen et al. (2024)	Moderate	Moderate	Direct	Moderate	Moderate	B
Chan et al. (2023)	Low	High	Direct	Precise	High	A
Randall et al. (2014)	Moderate	Moderate	Direct	Moderate	Moderate	B
Huang and Chen, (2021)	Moderate	Moderate	Direct	Moderate	Moderate	B
Carlson et al. (2015)	Moderate	Moderate	Direct	Moderate	Moderate	B
Morgan and Marroquín (2024)	Moderate	Moderate	Direct	Moderate	Moderate	B
Ghetti (2011)	Low	High	Direct	Moderate	High	A
Dingle and Fay (2017)	Moderate	Moderate	Direct	Moderate	Moderate	B
Dingle et al. (2016)	Moderate	Moderate	Direct	Moderate	Moderate	B
Kouhnavard et al. (2024)	Moderate	Moderate	Direct	Moderate	Moderate	B
Pasqualitto et al. (2025)	Low	Moderate	Direct	Moderate	Moderate	B
Nwokenna et al. (2022)	Moderate	Moderate	Direct	Moderate	Moderate	B
Sena Moore and Hanson-Abromeit (2015)	Moderate	Moderate	Direct	Moderate	Moderate	B
Cook et al. (2019)	Moderate	Moderate	Direct	Moderate	Moderate	B
Jackson (2023)	High	Low	Direct	Low	Low	C
Allocca (2025)	Moderate	Moderate	Direct	Moderate	Moderate	B
Chin and Rickard (2014b)	Moderate	Moderate	Direct	Moderate	Moderate	B
Saarikallio (2007)	Moderate	Moderate	Direct	Moderate	Moderate	B
Zemestani et al. (2023)	Low	Moderate	Direct	Moderate	Moderate	B
Randall et al. (2022)	Moderate	Moderate	Direct	Moderate	Moderate	B
Vidas et al. (2025)	Low	High	Direct	Moderate	High	A
Silverman (2021)	Moderate	Moderate	Direct	Moderate	Moderate	B
Robb et al. (2008)	Low	High	Direct	Moderate	High	A
Cao et al. (2025)	Low	Moderate	Direct	Moderate	Moderate	B
Chin and Rickard (2014a)	Moderate	Moderate	Direct	Moderate	Moderate	B
Köhler (2022)	Moderate	Moderate	Direct	Moderate	Moderate	B
Uhlig et al. (2018)	Low	High	Direct	Moderate	High	A
Uhlig et al. (2017)	Moderate	Moderate	Direct	Moderate	Moderate	B
Ferreri et al. (2021)	Moderate	Moderate	Direct	Moderate	Moderate	B
Song et al. (2024)	Moderate	Moderate	Direct	Moderate	Moderate	B
Park and Yoo (2026)	Moderate	Moderate	Direct	Moderate	Moderate	B
Zheng et al. (2024)	Moderate	Moderate	Direct	Moderate	Moderate	B
Saarikallio (2012)	Low	High	Direct	Precise	High	A
Völker (2021)	Moderate	Moderate	Direct	Moderate	Moderate	B
Zoteyeva et al. (2016)	Moderate	Moderate	Direct	Moderate	Moderate	B
Faran et al. (2021)	Moderate	Moderate	Direct	Moderate	Moderate	B
Siponkoski et al. (2022)	Low	High	Direct	Moderate	High	A
Li et al. (2025)	Moderate	Moderate	Direct	Moderate	Moderate	B
Fleszar-Pavlovic et al. (2024)	Moderate	Moderate	Direct	Low	Moderate	B
Aalbers et al. (2022)	Moderate	Moderate	Direct	Moderate	Moderate	B
Fachner et al. (2023)	Moderate	Moderate	Direct	Low	Moderate	B
Leipold and Loepthien (2015)	Moderate	Moderate	Direct	Moderate	Moderate	B
Bentley et al. (2023)	Low	High	Direct	Moderate	High	A
Julia et al. (2025)	Moderate	Moderate	Direct	Moderate	Moderate	B
Sanders (2026)	High	Low	Direct	Low	Low	C

## 4 Discussion

### 4.1 Summary of main findings

This meta-analysis synthesized evidence from 46 studies encompassing 3,740 participants across 11 countries, spanning randomized controlled trials, pre-post cohort designs, observational and experience-sampling studies, pilot trials, and exploratory or qualitative work. Across four synthesized outcome groups – general emotion and mood regulation, emotion and mood regulation in youth clinical populations, emotion and mood regulation in clinical and special populations, and stress reduction/coping/psychological well-being – pooled estimates were directionally consistent, ranging from 18.12 (95% CI 17.17–19.07) in youth clinical samples to 19.58 (95% CI 19.03–20.14) in clinical and special populations. This consistency across otherwise heterogeneous populations and intervention formats suggests that the association between music-based engagement and improved emotion regulation is not confined to a single demographic or delivery mode, but recurs across children, adolescents, university students, clinical inpatients, transplant recipients, veterans, and older adults alike.

Heterogeneity was moderate to substantial across subgroups (I^2^ = 51.5–80.4%), which is unsurprising given the breadth of designs, intervention formats (mobile applications, group and individual music therapy, mindfulness-based music programs, active engagement protocols, and self-directed listening), and outcome measurement approaches captured in this review. The GRADE appraisal reinforced this picture: only nine studies employing rigorous randomized designs with low risk of bias and high consistency achieved a high-quality (A) rating [1,3,8,22,24,28,34,38,44]; the majority of pre-post, observational, pilot, and cross-sectional studies were rated moderate quality (B); and exploratory case-study or purely qualitative designs were rated low quality (C) [16,46]. Taken together, the body of evidence supports a genuine but modest-to-moderate benefit of music-based interventions for emotion regulation, while signaling that confidence in the precise magnitude of that benefit should remain tempered until better-controlled, more homogeneous trials accumulate.

### 4.2 Contribution to the literature

Prior reviews in this space have tended to focus narrowly on a single population (e.g., adolescents, substance-use populations, or clinical cohorts) or a single delivery format (e.g., structured music therapy versus everyday music listening). By contrast, this review is, to our knowledge, among the first to bring together the full spectrum of music-based emotion-regulation research – from tightly controlled RCTs to naturalistic experience-sampling and qualitative work – within a single quantitative and appraisal framework. This contributes to the literature in several concrete ways.

First, by pooling effect estimates across four conceptually distinct but related outcome clusters, the review offers a comparative view of where the evidence base is strongest (general emotion regulation and clinical/special populations, with lower heterogeneity) and where it remains most inconsistent (stress/coping/well-being and youth clinical populations, with higher heterogeneity). This kind of cross-domain comparison has not previously been available and can help clinicians and researchers calibrate expectations about effect consistency by population and outcome type.

Second, the parallel application of design-appropriate risk-of-bias tools (Cochrane RoB 2 for RCTs, ROBINS-I for non-randomized intervention studies, and adapted AXIS criteria for cross-sectional and survey work) alongside GRADE quality-of-evidence ratings provides a methodological map of the field that prior narrative reviews have generally lacked. This map makes explicit which conclusions rest on high-certainty evidence (largely the RCT-based findings) and which rest on more provisional, hypothesis-generating evidence (the pre-post, cross-sectional, and qualitative literature).

Third, the inclusion of diverse intervention formats – digital/app-based delivery, group versus individual therapy, active engagement versus passive listening, and mindfulness-integrated protocols – within one synthesis begins to surface candidate moderators (delivery mode, dosage, active versus passive engagement) that future dose–response and dismantling trials could formally test. Importantly, these formats should not necessarily be interpreted as equivalent forms of “music therapy.” Music therapy represents a professional discipline delivered within a therapeutic relationship, whereas specific therapeutic models, techniques, music experiences, and delivery formats constitute distinct dimensions of an intervention. In this sense, the review functions not only as a summary of existing evidence but as a scaffold for the next generation of more targeted primary research.

### 4.3 Strengths

Several features strengthen the conclusions that can be drawn from this synthesis. The sample is large and geographically diverse, drawing on participants from Australia, China, Finland, Germany, Hong Kong, Italy, New Zealand, South Korea, Sweden, the United Kingdom, and the United States, and spanning an unusually wide age range (4.8 to 68.4 years) that includes preschoolers, school-age children, adolescents, university students, adults, and older adults. This breadth improves the generalizability of the overall directional finding, even where precision for any single subgroup remains limited.

The review also benefits from a transparent, PRISMA-guided selection process and from separating studies into four clinically meaningful outcome groups rather than forcing all 46 studies into a single, overly heterogeneous pooled estimate. Combining this subgroup structure with dual risk-of-bias and GRADE appraisal allowed the review to weight interpretive confidence appropriately: findings from the nine GRADE-A studies can be treated with greater confidence than the broader set of GRADE-B and GRADE-C studies, giving readers a graded rather than an all-or-nothing picture of the evidence.

Finally, by deliberately including non-randomized, observational, and qualitative studies alongside RCTs, the review captures ecologically valid, real-world uses of music for emotion regulation (such as self-directed everyday listening and experience-sampling studies) that would be excluded from an RCT-only synthesis, offering a more complete picture of how music-based emotion regulation operates both in controlled and naturalistic settings.

### 4.4 Limitations

This review has several limitations that temper the strength of its conclusions. Most importantly, statistical heterogeneity was moderate to substantial in every outcome group (I^2^ = 51.5–80.4%), reflecting genuine clinical and methodological diversity in participants, intervention protocols, durations (ranging from single sessions to 8 weeks), and outcome measures. Pooled estimates should therefore be interpreted as an indication of overall direction and approximate magnitude rather than as a precise, generalizable effect size.

A substantial proportion of the included studies were non-randomized (pre-post cohorts, single-group pilot designs, cross-sectional surveys, and exploratory or qualitative work), and risk-of-bias appraisal using ROBINS-I and adapted AXIS criteria rated several of these as carrying moderate-to-serious risk, most often due to the absence of a control or comparison group, unaddressed confounding, and reliance on convenience samples such as university students. Even among the RCTs, Cochrane RoB 2 appraisal found that most fell into the “some concerns” category, chiefly reflecting unclear or incompletely reported randomization and allocation procedures and limited detail on outcome-assessor blinding.

Sample sizes in many of the included pilot and feasibility studies were small (as low as *n* = 10–40), limiting statistical power at the level of individual studies and increasing the influence of any single small trial on subgroup pooled estimates. Outcome measurement also varied considerably: most studies relied on self-report instruments, and depression, anxiety, and emotion-regulation constructs were not always assessed with the same validated scales across studies, introducing measurement heterogeneity that likely contributes to the observed statistical heterogeneity. Additionally, most studies assessed outcomes only immediately post-intervention, so the review cannot speak to the durability of emotion-regulation gains over the medium or long term. Finally, while a funnel plot was examined, formal small-study/publication-bias testing (e.g., Egger’s regression) was not exhaustively reported for every subgroup, so the possibility that unpublished null or negative findings are underrepresented in this literature cannot be fully excluded.

The interventions used in the meta-analysis also differed significantly in terms of their theoretical base, the model, techniques, musical experiences, implementation methods, and qualifications required for professionals. Specifically, the term “music therapy” cannot be equated to a wider range of activities that include music listening, singing, playing an instrument, and improvisation. While these activities can be performed in the framework of music therapy, they can also be practiced independently or even outside of the music therapy practice. Individual, group, and app interventions are simply different implementation methods rather than separate schools of therapy. The diversity in conceptualization of music therapy may affect intervention outcomes and make a comparison between the studies difficult. That is why the results obtained must be understood as evidence of the efficacy of a wide range of music interventions.

### 4.5 Future prospects

Building on these findings and limitations, several priorities emerge for future research. Larger, adequately powered, multi-site RCTs with pre-registered protocols, concealed allocation, and blinded outcome assessment are needed, particularly in the youth clinical and stress/coping/well-being domains where heterogeneity was highest, and evidence quality most often fell short of GRADE-A. Greater standardization of outcome measurement – for example, consistent use of validated instruments such as the Emotion Regulation Questionnaire (ERQ) or the Difficulties in Emotion Regulation Scale (DERS) alongside standardized depression and anxiety measures – would substantially reduce measurement-driven heterogeneity and improve comparability across future syntheses.

Dismantling and dose–response trials are also needed to isolate which components of music-based interventions drive benefit: active engagement versus passive listening, group versus individual delivery, therapist-led versus self-directed/app-based formats, and shorter versus longer intervention durations. Given the promising but still preliminary evidence from digital and eHealth delivery formats identified in this review, trials evaluating scalability, adherence, and cost-effectiveness of app-based or telehealth music interventions represent a particularly high-value direction, especially for populations with limited access to in-person music therapy.

Future studies should also prioritize long-term follow-up to determine whether emotion-regulation gains are maintained beyond the immediate post-intervention window. They should extend recruitment beyond convenience samples of university students to more representative community, clinical, and cross-cultural populations. Finally, incorporating objective or physiological correlates of emotion regulation (e.g., EEG, neuroimaging, or psychophysiological indices, as piloted in a small number of included studies) alongside self-report outcomes would help clarify the mechanisms through which music-based interventions influence emotional processing, and would strengthen the evidentiary base beyond the self-report-dominated literature synthesized here.

## 5 Conclusion

This meta-analysis of 46 studies and 3,740 participants indicates that music-based interventions – spanning mobile applications, group and individual music therapy, mindfulness-based music programs, active music engagement, and self-directed listening – are consistently associated with improved emotion regulation and reduced depressive and anxiety symptoms across general, youth clinical, and clinical/special populations, as well as in stress-reduction and coping contexts. Pooled estimates were directionally consistent across all four outcome groups (18.12–19.58), though moderate-to-substantial heterogeneity (I^2^ = 51.5–80.4%) and a predominance of moderate-quality (GRADE B) evidence indicate that these effects should be interpreted as promising rather than conclusive. The strongest evidence, drawn from nine GRADE-A randomized controlled trials, supports a genuine benefit of structured, well-controlled music-based interventions. At the same time, the larger body of pre-post, observational, cross-sectional, and qualitative studies offers converging but methodologically weaker support. Taken together, the evidence base is sufficiently consistent to justify continued clinical and educational use of music-based approaches for emotion regulation, but not yet robust enough to specify optimal intervention type, dosage, or duration with precision. Closing this gap will require larger, adequately powered, pre-registered RCTs with standardized outcome measures and longer follow-up, alongside continued exploration of the naturalistic and technology-delivered formats that make music-based emotion regulation broadly accessible in everyday life.

## Data Availability

The raw data supporting the conclusions of this article will be made available by the authors, without undue reservation.
